# Profiling bile acid composition in bile from mice of different ages and sexes

**DOI:** 10.3389/fphys.2025.1626215

**Published:** 2025-09-01

**Authors:** Jiajian Liu, Qing Zhao, Chun Qu, Kun Ge, Yang Li, Wei Jia, Aihua Zhao

**Affiliations:** Center for Translational Medicine, Shanghai Sixth People’s Hospital Affiliated to Shanghai Jiao Tong University School of Medicine, Shanghai, China

**Keywords:** bile, bile acid, sex, age, mouse

## Abstract

**Introduction:**

Bile BA profiling is important for understanding the pathogenetic mechanisms underlying gallbladder and gastrointestinal diseases. Until now, bile BAs have been explored mainly in adult male mice. However, sex- and age-related variations in the bile BA composition of normal experimental mice remain poorly characterized.

**Methods:**

BAs in bile from healthy, age-and sex-matched mice (8-week-old and 60-week-old) were quantified using ultra-performance liquid chromatography coupled with triple-quadrupole mass spectrometer (UPLC-TQ-MS). The bile BA profiles were comprehensively compared across different ages and sexes, along with comparisons within the same sex at different ages.

**Results:**

Total BAs (TBAs) in female mice were higher than those in male mice at both ages, although total BAs and BA profiles were similar between the two ages. High concentrations of BAs contributed significantly to the observed sex differences at both ages. Notably, both high- and low-concentration BAs were regulated with increasing age in male mice, while only low-concentration BAs were modulated with age in female mice. A remarkable sex difference in total cholesterol (TC) was also observed, along with a significant negative association between TC and BA profiles.

**Conclusion:**

The findings demonstrate that bile BA profiles differ markedly with both sex and physiological age in commonly used normal laboratory mice, suggesting the need to consider for physiological differences related to sex and age when selecting suitable animals for pharmaceutical research and for mechanistic studies.

## 1 Introduction

Bile is a digestive fluid synthesized from cholesterol in the liver, stored in the gallbladder, and then released into the small intestine to aid digestion and absorption of dietary lipids (Ahmed, 2022; Podgorski et al., 2023; Bozadjieva-Kramer et al., 2024; Fuchs et al., 2013). The main constituents of bile include bile acids (BAs), fatty acids, amino acids, phospholipids, cholesterol, and bilirubin, along with other trace metals and ions (Dosch et al., 2019).

BAs, which are the major components of bile, act as signaling molecules, binding to BA membrane receptors or nuclear receptors in various tissues to regulate lipid and glucose metabolism (Jia et al., 2024; Jia et al., 2021; Matz-Soja, 2020). Notably, tauroursodeoxycholic acid (TUDCA) reduces age-related hyperinsulinemia in mice (Zangerolamo et al., 2022), mediates insulin sensitivity in obese mice (Zhang et al., 2022), ameliorates diabetic kidney disease in streptozotocin-induced diabetic mice (Sankrityayan et al., 2023), attenuates inflammatory responses, and modulates intestinal microbiota composition to alleviate the progression of non-alcoholic fatty liver disease (NAFLD) induced by a high-fat diet in mice (Wang et al., 2018). Chenodeoxycholic acid (CDCA) improves acute pancreatitis by mediating the farnesoid X receptor (FXR) (Zhu et al., 2023). Our previous research has also revealed that hyocholic acid (HCA) species, including HCA, hyodeoxycholic acid (HDCA), and their glycine and taurine conjugates, play a crucial role in maintaining blood sugar homeostasis. This is achieved by simultaneously activating two BA receptors, FXR and G protein-coupled receptor 5 (TGR5), to regulate the production and secretion of glucagon-like peptide-1 (GLP-1) (Zheng J. et al., 2021). Additionally, HDCA and glyco-HDCA (GHDCA) exert therapeutic effects on NAFLD in multiple mouse models. The effects are mediated by inhibiting FXR to activate hepatic alternative BA synthesis and increasing the abundance of probiotic species to inhibit hepatic classic BA synthesis (Kuang et al., 2023; Zhong et al., 2023). Collectively, these two signaling pathways contribute to enhanced lipid catabolism.

Rodents, particularly mice, are commonly used as experimental animals to investigate the pathophysiological activities of pharmaceuticals and molecular mechanisms in laboratories (Li and Dawson, 2019; Mariotti et al., 2018). Previous studies on BA profiles have predominantly focused on serum, feces, liver, and intestine (Ma et al., 2020; Ma et al., 2021). Bile BAs have been explored only in adult male mice (Alnouti et al., 2008; Zheng X. et al., 2021); investigation into sex- and age-related variations in BA composition of normal mouse bile remains limited. Bile BAs, to some extent, can reflect the composition of BAs in the liver and upper gastrointestinal tract. Investigating the characteristics of BA composition in the gallbladder under physiological conditions is essential for understanding the pathogenic mechanisms underlying gallbladder and gastrointestinal diseases. Moreover, a comprehensive comparison of gallbladder BA profiles across sexes and ages—together with an identification of age-related changes within each sex—is crucial for evaluating drug metabolism and improving the precision of therapeutic interventions.

## 2 Materials and methods

### 2.1 Chemicals and reagents

BA standards, including cholic acid (CA) (Cat. No. C1900-015), glycocholic acid (GCA) (Cat. No. C1925-000), taurocholic acid (TCA) (Cat. No. C1965-000), CDCA (Cat. No. C0940-000), glycochenodeoxycholic acid (GCDCA) (Cat. No. C0962-000), taurochenodeoxycholic acid (TCDCA) (Cat. No. C0990-000), α-muricholic acid (αMCA) (Cat. No. C1890-000), β-muricholic acid (βMCA) (Cat. No. C1895-000), tauro α-muricholic acid (TαMCA) (Cat. No. C1893-000), tauro β-muricholic acid (TβMCA) (Cat. No. C1899-000), ursodeoxycholic acid (UDCA) (Cat. No. C1020-000), TUDCA (Cat. No. C1052-000), taurodeoxycholic acid (TDCA) (Cat. No. C1162-000), taurohyocholic acid (THCA) (Cat. No. C1887-000), HDCA (Cat. No. C0860-000), taurohyodeoxycholic acid (THDCA) (Cat. No. C0892-000), ω-muricholic acid (ωMCA) (Cat. No. C1888-000), and tauro ω-muricholic acid (TωMCA) (Cat. No. C1889-000) were purchased from Steraloids Inc. (Newport, RI, United States). The stable isotope-labeled BAs used for internal standards (ISs), including cholic acid-d_4_ (CA-d_4_) (Cat. No. D-2452), glycocholic acid-d_4_ (GCA-d_4_) (Cat. No. D-3878), taurocholic acid-d_4_ (TCA-d_4_) (Cat. No. D-8089), chenodeoxycholic acid-d_4_ (CDCA-d_4_) (Cat. No. D-2772), glycochenodeoxycholic acid-d_4_ (GCDCA-d_4_) (Cat. No. D-5673), taurochenodeoxycholic acid-d_4_ (TCDCA-d_4_) (Cat. No. D-8183), ursodeoxycholic acid-d_4_ (UDCA-d_4_) (Cat. No. D-3819), and taurodeoxycholic acid-d_4_ (TDCA-d_4_) (Cat. No. D-8182) were obtained from CDN Isotopes Inc. (Quebec, Canada); hyocholic acid-d_5_ (HCA-d_5_) (Cat. No. DLM-10628) was purchased from Cambridge Isotope Laboratories Inc. (CIL) (Tewksbury, United States), and hyodeoxycholic acid-d_5_ (HDCA-d_5_) (Cat. No. TRC-H998102) was obtained from LGC Standards-Toronto Research Chemicals (TRC) (Toronto, Canada). All standards had a purity higher than 98%. The LC-MS grade methanol (MeOH) and acetonitrile (ACN) were purchased from Thermo Fisher Scientific (Waltham, MA, United States). Formic acid was obtained from Sigma-Aldrich (Missouri, United States). Ultrapure water (18.2 MΩ⋅cm) was produced using the Milli-Q Purification System (Merck Millipore, Darmstadt, Germany) in our laboratory. Serum total cholesterol (TC) was measured using a commercial kit (S03042, Rayto Life and Analytical Sciences Co., Ltd., Shenzhen, China).

### 2.2 Sample collection

Mouse bile was collected from healthy, age- and sex-matched C57BL/6J mice (Shanghai SLAC Laboratory Animal Co., Ltd., Shanghai, China). The study included 24 mice (12 male and 12 female) at 8 weeks of age and 28 mice (14 male and 14 female) at 60 weeks of age. All mice had free access to chow and tap water *ad libitum*. The formula of diet chow (from Trophic Animal Feed High-tech Co., Ltd., China) is shown in Table 1. No dietary intervention was implemented until the fasting period was initiated for sample collection the following morning. After a 12-h fast, the mice were euthanized, and the gallbladder was extracted to obtain bile samples. The samples were stored at −80 °C until analysis.

**TABLE 1:** Composition of normal diet chow.

Component	Control chow diet (g)
Casein	191.800
Corn starch	471.600
Dextrin	126.600
Sucrose	73.400
Soybean oil	40.100
Lard	\
Cellulose	48.000
Mineral	33.600
Vitamin	9.600
L-Cystine	2.900
Choline chloride	2.400
Tertiary butylhydroquinone	0.008
Total	1000.000
Proteins	19.000%
Carbohydrates	71.000%
Lipids	10.000%

All mouse experiments were conducted in accordance with standard operating procedures and were approved by the Sixth People’s Hospital affiliated with Shanghai Jiao Tong University School of Medicine (ethics approval number: No. 2021-0110).

### 2.3 Pooled sample

Pooled samples were prepared by combining aliquots of bile from each animal and treating them with activated charcoal to remove endogenous BAs.

### 2.4 Standard calibration curve

All standards were spiked into the pooled samples, and the calibration curves were subsequently established with IS adjustment. According to the actual content of the BAs in mouse bile, the standard concentrations for the calibration curves were divided into two groups: a low-concentration group with concentrations at 1, 2, 5, 10, 20, 50, 100, 200, and 500 nmol/L (including GCA, CDCA, GCDCA, αMCA, UDCA, HDCA, and THCA) and a high-concentration group with concentrations at 10, 20, 50, 100, 200, 500, 1,000, 2,000, and 5,000 nmol/L (including CA, TCA, TCDCA, βMCA, TαMCA, TβMCA, TUDCA, TDCA, THDCA, ωMCA, and TωMCA).

### 2.5 Instrument conditions

All analyses were performed on a Waters ACQUITY Ultra-Performance Liquid Chromatography System coupled with a Waters Xevo Triple Quadrupole Mass Spectrometer, controlled using MassLynx V4.2 software (Waters, United States). BA separations were performed on a CORTECS® C18 Column (2.1 × 100 mm, 1.6 μm, Waters) with a CORTECS® C18 VanGuard™ Pre-Column (2.1 × 5 mm, 1.6 μm, Waters) at 45 °C. The mobile phase consisted of ultrapure water (A) and ACN: MeOH (9:1, v/v) (B), each containing 0.01% formic acid at an eluting rate of 0.45 mL/min. The gradient was maintained as follows: 0–1.0 min (5%–20% B), 1.0–2.0 min (20%–25% B), 2.0–4.5 min (25% B), 4.5–5.0 min (25%–30% B), 5.0–6.5 min (30% B), 6.5–8.0 min (30%–35% B), 8.0–15.0 min (35%–65% B), 15.0–16.0 min (65%–100% B), 16.0–18.0 min (100% B), 18.0–18.1 min (100%–5% B), and 18.1–20.0 min (5% B). The injection volume was 5 μL.

**FIGURE 1:**
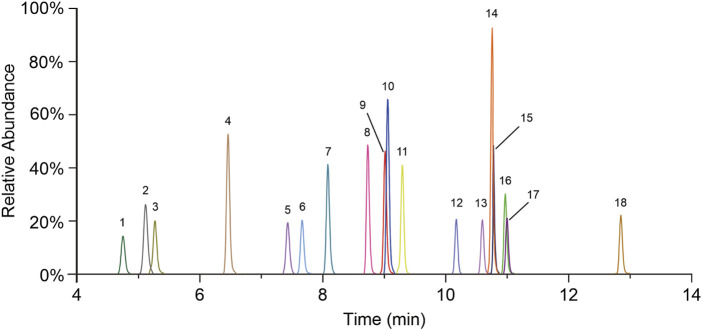
MRM chromatograms of all quantitative BAs. 1. TωMCA, 2. TαMCA, 3. TβMCA, 4. THCA, 5. TUDCA, 6. THDCA, 7. TCA, 8. ωMCA, 9. αMCA, 10. GCA, 11. βMCA, 12. TCDCA, 13. TDCA, 14. CA, 15. UDCA, 16. HDCA, 17. GCDCA, and 18. CDCA.

Mass data were acquired in multiple reaction monitoring (MRM) mode under negative ionization, using an electrospray ionization (ESI) source. The mass spectrometer source settings are as follows: capillary voltage, 3.0 KV; desolvation gas flow, 1,000 L/h; desolvation gas temperature, 500 °C; and collision gas flow, 0.15 mL/min. The quantitative mass pair (m/z) and collision energy (eV) are presented in Table 2, and the MRM chromatographic peaks of all BAs are shown in Figure 1.

**TABLE 2:** Validation parameters of the quantitative BA method.

Compound	Mass pair (m/z)	Collision energy (eV)	R^2^	Linear range (nmol/L)	LOD (nmol/L)	LOQ (nmol/L)	Intra-day precision			Inter-day precision			Recovery			ME (%) (n = 3)
							(RSD%) (n = 3)			(RSD%) (n = 9)			(%) (n = 3)			
							STD-L	STD-M	STD-H	STD-L	STD-M	STD-H	STD-L	STD-M	STD-H	
CA	407.3/407.3	10	0.999	10–5000	0.2	0.5	0.84	2.07	1.28	11.63	9.58	3.97	113.89	92.43	101.07	0.00
GCA	464.4/73.9	48	0.998	1–500	0.2	1.0	9.43	2.51	1.03	12.48	3.46	8.27	105.51	113.21	102.47	0.14
TCA	514.4/79.9	70	0.999	10–5000	0.5	1.0	2.07	8.21	3.61	6.12	8.50	4.48	112.59	106.06	104.96	0.10
CDCA	391.3/391.3	10	0.998	1–500	0.5	1.0	2.67	0.94	1.30	8.50	4.31	3.92	101.41	109.60	110.63	0.02
GCDCA	448.4/73.9	40	0.999	1–500	0.2	0.5	12.00	2.10	2.20	15.65	4.98	5.61	97.63	98.54	95.11	0.03
TCDCA	498.4/79.9	75	0.996	10–5000	1.0	2.0	1.50	5.62	7.02	12.01	6.84	6.62	112.44	108.97	107.61	0.15
αMCA	407.3/407.3	10	0.999	1–500	0.5	1.0	12.48	3.05	1.97	13.70	8.02	3.18	99.85	97.71	114.38	−0.05
βMCA	407.3/407.3	10	0.996	10–5000	0.5	1.0	3.98	4.05	1.55	14.01	7.56	5.92	102.59	96.41	112.17	−0.07
TαMCA	514.4/79.9	75	0.999	10–5000	0.2	0.5	3.07	0.93	1.75	11.92	3.03	5.21	87.55	101.13	97.45	0.04
TβMCA	514.4/79.9	75	0.998	10–5000	0.1	0.2	3.50	2.34	1.62	10.26	5.85	4.15	101.02	97.33	97.94	−0.15
UDCA	391.3/391.3	10	0.999	1–500	0.5	1.0	3.99	1.48	1.42	10.38	5.02	3.39	102.10	104.89	113.59	−0.02
TUDCA	498.4/79.9	75	0.996	10–5000	1.0	2.0	14.42	4.40	8.84	12.86	8.34	7.54	109.02	111.43	99.02	−0.14
TDCA	498.4/79.9	75	0.997	10–5000	1.0	2.0	4.16	1.63	9.56	5.79	9.40	9.95	114.44	112.09	99.30	0.19
THCA	514.4/79.9	70	0.996	1–500	0.5	1.0	2.53	0.80	1.00	8.61	5.51	6.25	107.63	92.00	85.96	−0.06
HDCA	391.3/391.3	10	0.997	1–500	0.2	0.5	4.71	2.40	2.96	9.41	7.24	11.13	91.45	109.61	91.19	0.07
THDCA	498.4/79.9	75	0.999	10–5000	0.5	1.0	8.85	6.61	9.20	12.60	13.23	12.20	98.28	100.75	103.17	−0.15
ωMCA	407.3/407.3	10	0.999	10–5000	0.2	0.5	3.79	3.84	2.10	10.38	7.51	4.93	110.61	96.92	106.20	−0.04
TωMCA	514.4/79.9	10	0.999	10–5000	0.5	1.0	8.33	4.36	2.59	10.85	6.48	5.61	106.17	96.40	98.21	0.06

### 2.6 Validation of the BA quantitative method

To ensure the accuracy and reliability of the quantitative BAs, we validated linearity, limit of detection (LOD), limit of quantification (LOQ), precision, accuracy, and matrix effect (ME). The results of the linear equations (R^2^ > 0.996), LOD (0.2–1.0 nmol/L), LOQ (0.5–2.0 nmol/L), RSD of intra-day precision (0.80%–14.42%), RSD of inter-day precision (3.03%–15.65%), accuracy (85.96%–114.44%), and ME (−0.15% to 0.19%) are presented in Table 2. All these results implied that the established BA quantification method met the requirements for biological sample quantification.

### 2.7 Sample preparation

Mouse bile was diluted 1000-fold with ultrapure water before preprocessing. Each 20 μL diluted bile was mixed with 60 μL of a MeOH: water (1:1, v/v) solution containing 10 ISs at concentrations of 50 nmol/L each. The resultant mixtures were vortexed for 5 min and subsequently centrifuged at 13,200 rpm and 4 °C for 10 min. A 60 μL aliquot of supernatant was taken for MS analysis. For high concentrations of TCA, TβMCA, and TωMCA in mouse bile, bile was further diluted 50-fold with a MeOH: water (1:1, v/v) solution to fit the linearity ranges.

### 2.8 Statistical analysis

Data acquisition was executed using MassLynx software version 4.2, and quantification analysis was conducted using the TargetLynx version 4.2 (Waters, Milford, MA).

The data were expressed as the mean ± SEM. The pairwise comparisons were performed using the Mann–Whitney test. A *p*-value (*p* < 0.05) was defined as statistically significant. All the figures were generated using GraphPad Prism software, version 8.3.0 (GraphPad Software, Inc.). Pearson’s correlation analysis was performed to assess the associations between serum TC and both total BAs (TBAs, the sum of all individual BAs detected) and primary conjugated BAs (PCBAs), with significance set at *p* < 0.05.

## 3 Results

**FIGURE 2:**
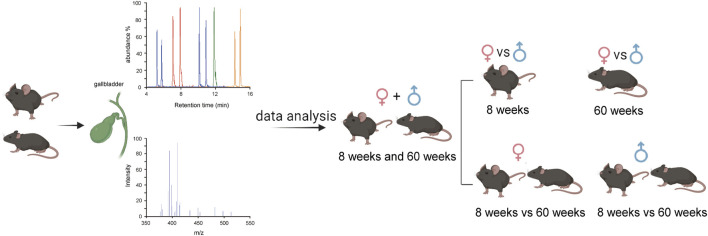
Flowchart of the bile BA analysis.

We conducted a comprehensive analysis of BA compositions in the bile of C57BL/6J mice, considering two age groups (8-week-old and 60-week-old) and both sexes. The flowchart of the analysis is depicted in Figure 2.

### 3.1 BA compositions at different ages

**FIGURE 3:**
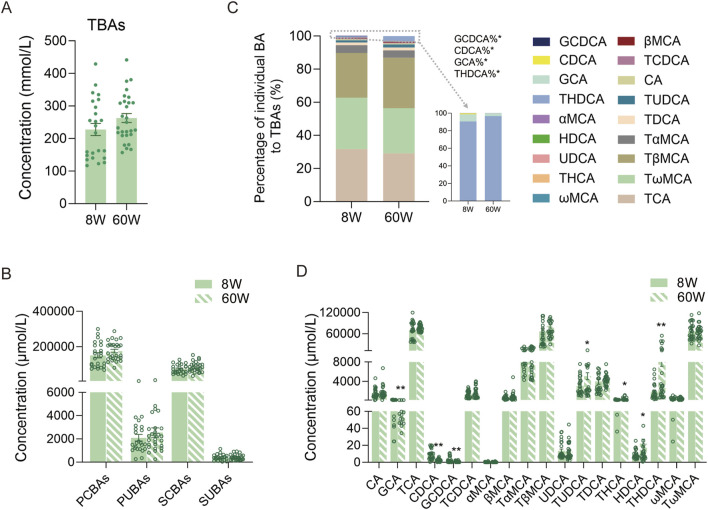
Comparison of TBA levels and BA composition in 8-week-old and 60-week-old mice. **(A)** TBA levels at the two ages. **(B)** PCBA, PUBA, SCBA, and SUBA levels at the two ages. **(C)** BA percentage compositions at the two ages. **(D)** Individual BA levels at the two ages. TBAs, total BAs, the sum of all individual BAs detected; 8W, 8-week; 60W, 60-week; PCBAs: TCA, GCA, TCDCA, GCDCA, TαMCA, TβMCA, and TUDCA; PUBAs: CA, CDCA, αMCA, βMCA, and UDCA; SCBAs: TωMCA, TDCA, THCA, and THDCA; SUBAs: ωMCA and HDCA; *p < 0.05 and **p < 0.01; sample size: n = 24 for 8W and n = 28 for 60W.

We first compared the TBAs in all mice at 8 weeks and 60 weeks. There were no significant differences between them (Figure 3A). We further analyzed PCBAs, primary unconjugated BAs (PUBAs), secondary conjugated BAs (SCBAs), and secondary unconjugated BAs (SUBAs) in the two age groups of mice. The results also showed that there were no significant differences in any group of BAs (Figure 3B). The BA profiles predominantly consisted of PCBAs (including TCA, GCA, TCDCA, GCDCA, TαMCA, TβMCA, and TUDCA) and SCBAs (including TωMCA, TDCA, and THDCA), which accounted for more than 98% of BAs in the bile of both age groups of mice. Among them, TCA, TβMCA, and TωMCA comprised more than 85% (Figure 3C). Among the BA profiles, only the percentages of THDCA, GCA, CDCA, and GCDCA displayed differences between the two age groups of mice (Figure 3C). For the individual BA concentrations, the levels of primary BAs, including GCA, CDCA, and GCDCA, were significantly higher in 8-week-old mice than in 60-week-old mice. In contrast, the levels of primary BA TUDCA and secondary BAs, including HDCA, THDCA, and THCA, were significantly higher in 60-week-old mice than in 8-week-old mice (Figure 3D).

### 3.2 BA compositions in different sexes

**FIGURE 4:**
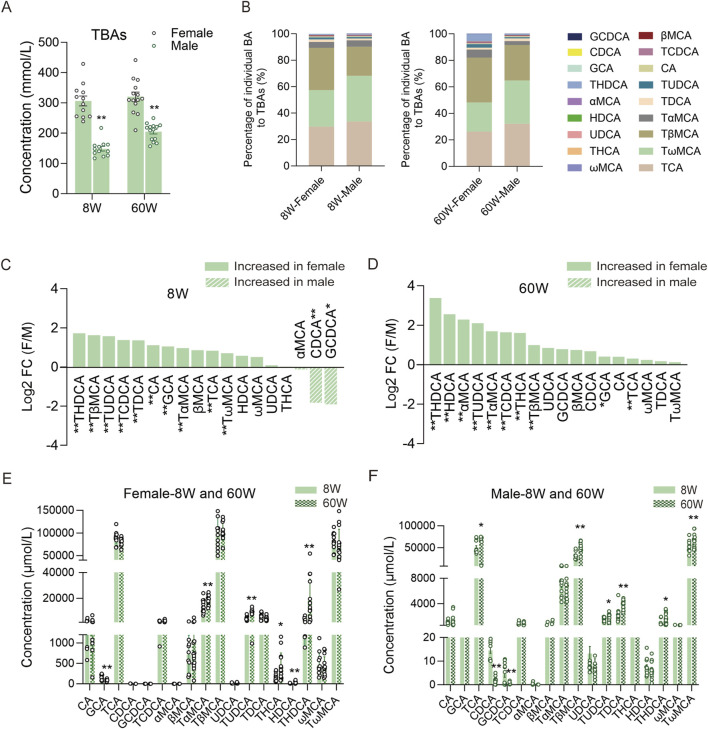
Comparison of BA compositions between different sexes and within the same sex across the two ages. **(A)** TBA levels of male and female mice at 8 and 60 weeks. **(B)** BA percentage compositions of male and female mice at the two ages. **(C)** FC (female to male) in 8-week-old mice. **(D)** FC (female to male) in 60-week-old mice. **(E)** Individual BA concentrations in female mice between the two ages. **(F)** Individual BA concentrations in male mice between the two ages. FC, fold change; F, female; M, male; 8W, 8-week; 60W, 60-week; *P < 0.05 and **P < 0.01; sample size: n = 12 for female-8W, n = 12 for male-8W, n = 14 for female-60W, and n = 14 for male-60W.

To comprehensively understand bile BAs in male and female mice, we further analyzed the BAs in different sexes. As a result, we observed that TBAs in female mice were markedly higher than those in male mice at both ages (Figure 4A), and their BA profiles in percentages also differed markedly (Figure 4B). In 8-week-old mice, TβMCA accounted for 32.10% of TBAs in female mice, significantly higher than the 22.01% in male mice, whereas TωMCA accounted for 34.54% in male mice, significantly higher than the 27.63% in female mice. Another predominant BA in bile, TCA, showed no significant difference between male and female mice of this age. In 60-week-old mice, the percentage of TβMCA increased to 26.83% in male mice and 33.96% in female mice, while the percentage of TωMCA decreased to 32.65% in male mice and 21.99% in female mice; these two BAs maintained significant differences. However, at this age, the percentage of TCA in male mice decreased to 32.12%, significantly higher than the 26.18% in female mice. When comparing the fold changes (FC: female to male mice) of individual BAs in 8-week-old mice, we found that most BAs were higher in female mice than in male mice, except for GCDCA, CDCA, and αMCA, which were higher in male mice than in female mice. For individual BAs, except for βMCA, HDCA, ωMCA, UDCA, THCA, and αMCA, the remaining BAs displayed differences between the two sexes (Figure 4C). In 60-week-old mice, all of the BAs were higher in female mice. Specifically, the concentrations of THDCA, HDCA, αMCA, TUDCA, TαMCA, TCDCA, THCA, TβMCA, GCA, and TCA were significantly higher in female mice than in male mice (Figure 4D). Additionally, when we compared BA compositions within the same sex at different ages, we found that only some relatively low-concentration BAs, such as TαMCA, TUDCA, THCA, THDCA, HDCA, and GCA, changed significantly in female bile with increasing age (Figure 4E). However, high concentrations of BAs, including TCA, TβMCA, and TωMCA, along with some low concentration BAs, including TUDCA, TDCA, THDCA, GCDCA, and CDCA, changed significantly in male bile BAs (Figure 4F).

### 3.3 Correlation of BAs with serum TC

**FIGURE 5:**
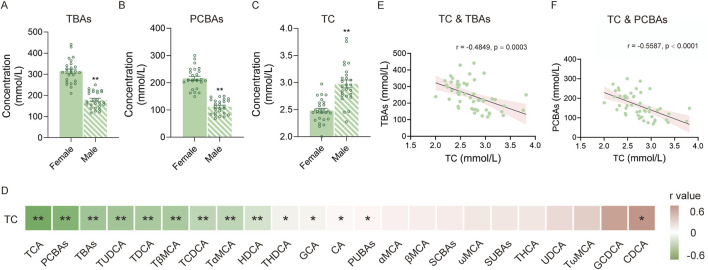
Correlation of BAs with TC. **(A)** TBA levels of female and male mice. **(B)** PCBA levels of female and male mice. **(C)** TC level of female and male mice. **(D)** Heatmap of correlations between different BAs and TC. **(E)** Correlation between TC and TBAs. **(F)** Correlation between TC and PCBAs. *P < 0.05 and **P < 0.01; sample size: n = 26 for female and n = 26 for male.

As TBA and PCBA levels significantly differed between male and female mice (Figures 5A,B), we further measured serum TC in all mice across two age groups. The results showed that TC levels were significantly lower in female mice than in male mice (Figure 5C). Furthermore, we performed correlation analysis between BA profiles and TC levels (Figure 5D). As a result, we observed that half of the BA individuals, TBAs, PCBAs, and PUBAs were related to serum TC levels, whereas CDCA was positively correlated with TC. Notably, TBAs and PCBAs showed a significant negative correlation with TC (Figures 5E,F).

## 4 Discussion

In this study, to accurately quantify each BA in bile samples, we analyzed each sample twice at concentrations differing by 50-fold, based on our established quantitative method. This approach ensured a dynamic range covering over 10^6^ times in the sample concentration. Additionally, we divided the calibration curves into a low-concentration group and a high-concentration group to accommodate the quantitative analysis of BAs in mouse bile. As a result, conjugated BAs, including both primary and secondary conjugated BAs, constituted more than 98% of the TBAs in mouse bile, consistent with previous studies (Alnouti et al., 2008; Zheng J. et al., 2021; Huang et al., 2011).

Upon comparing the BA profiles of normal 8-week-old and 60-week-old C57BL/6J mice, we found that although TBA levels and BA profiles were similar across the two ages, there were distinct differences between male and female mice within each age group. BA levels were generally higher in female mice than in male mice. Notably, our data revealed that THDCA levels in bile from normal mice were significantly higher in female mice than in male mice in both the 8-week and 60-week groups, and this trend became more pronounced with increasing age, reaching an over 10-fold difference in the 60-week-old group. This observed result may be consistent with the lower incidence of NAFLD in women, which is attributed to the higher levels of THDCA in women than in men, as reported in previous findings (Nagral et al., 2022). Additionally, we observed that, in female mice, the relatively low concentrations of several BAs, including GCA, TαMCA, TUDCA, THCA, HDCA, and THDCA, changed with increasing age. In male mice, both high-concentration BAs (TCA, TβMCA, and TωMCA) and low-concentration BAs (CDCA, GCDCA, TUDCA, TDCA, and THDCA) changed with increasing age. These results indicated that female mice may maintain a relative homeostasis of bile compositions, whereas male mice undergo more significant changes than female mice with increasing physiological age. This may be explained by the significant individual variation in the effects of the estrous cycle and hormones on BA concentrations or composition, which can conceal BA changes with increasing age (Yan et al., 2022). Sex- and age-related differences in BA composition may largely result from the regulatory effects of sex hormones in BA synthesis, metabolism, and transport. Published studies show that estrogen enhances the activity of BA-synthetic enzymes (CYP7A1 and CYP8B1), represses metabolic enzymes (CYP3A1, CYP3A11, and SULT2A1), downregulates BSEP and MRP2 expressions, and inhibits NTCPs and OATPs, thereby increasing intrahepatocellular BA levels and decreasing bile flow (Zu et al., 2021; Yang et al., 2019; Wang et al., 2019; Yamamoto et al., 2006). The resulting increase in hepatic synthesis and decrease in biliary secretion into the intestine may potentially elevate BA levels in female mice, inducing sex differences in bile BA profiles. Our results showed significantly higher TBA levels in female mice than in male mice, which may be attributed to the physiologically higher estrogen levels in normal reproductive-age female mice. Notably, when comparing ages, although 60-week-old mice are approaching the end of their reproductive period, TBA levels did not decrease significantly, likely because the published report indicates no significant difference in estrogen levels between young and 14-month-old mice (Nelson et al., 1981). Additionally, these sex differences in BA pool size and composition may be related to the significantly higher incidence of gallbladder diseases, such as cholelithiasis and PBC, in women than in men (Petrov et al., 2020; Chen et al., 2020).

Bile BAs come from hepatic BAs, which consist of newly synthesized BAs and those reabsorbed via enterohepatic circulation. Consequently, the composition of bile BAs can reflect the BA profiles of the liver and upper gastrointestinal tract to some extent. For example, altered bile BA composition has been observed in mice with liver injury (Zhou et al., 2022) and in non-alcoholic steatohepatitis (NASH) mice (Gillard et al., 2022), indicating that bile BAs are closely related to liver health status. Additionally, BA profiles differ substantially across bile, serum, and liver, each exhibiting distinct characteristics. TBA levels increase approximately 300-fold from plasma to the liver and a further approximately 175-fold from the liver to bile (Alnouti et al., 2008). Our studies indicate that conjugated BAs, including both PCBAs and SCBAs, account for 98.80% of TBAs, with PCBAs comprising 65.86% and SCBAs comprising 32.94%. Similar to bile BAs, hepatic conjugated BAs range from 45.08% to 98.70% of TBAs, with PCBAs contributing 39.80%–93.39% and SCBAs contributing 4.78%–26.91%, based on the published reports (Alnouti et al., 2008; Suga et al., 2019; Hermeling et al., 2025; Garcia-Canaveras et al., 2012). In contrast to bile and hepatic BA profiles, serum exhibits a markedly different distribution, with PCBAS, SCBAs, PUBAs, and SUBAs accounting for approximately 21.94%, 37.67%, 11.93%, and 28.05%, respectively, according to our previous report (Zheng et al., 2024). These results align with the reported gradient of primary BAs: highest in bile (approximately 95%), intermediate in the liver (approximately 84%), and lowest in plasma (approximately 66%) (Choudhuri and Klaassen, 2022).

Furthermore, our observed results demonstrated a negative correlation between BAs and TC. BAs provide an important excretory pathway for cholesterol metabolism, with more than 50% of cholesterol catabolism being achieved through BA synthesis (Russell, 2009). The lower TC levels in female mice may thus be attributed to their higher BA levels than those in male mice. This study has validated the link between TC and BA levels and cholesterol metabolism. These findings further emphasize the importance of considering sex differences when selecting suitable animals for experimental models related to BA metabolism.

## 5 Conclusion

Analysis of mouse bile BAs shows significant sex differences in both 8-week-old and 60-week-old mice. Murine BAs, including TβMCA and TωMCA, play important roles in these differences. Within the same sex at different ages, predominant BAs and low-concentration BAs display marked differences in male bile, whereas only low-concentration BAs exhibit changes in female mice with increasing age. These differences in laboratory mice must be considered when selecting suitable mice for pharmaceutical activity research and investigating molecular mechanisms.

## Data Availability

The original contributions presented in the study are included in the article/supplementary material, further inquiries can be directed to the corresponding author.
